# Real-Time Analysis of Potassium in Infant Formula Powder by Data-Driven Laser-Induced Breakdown Spectroscopy

**DOI:** 10.3389/fchem.2018.00325

**Published:** 2018-07-31

**Authors:** Da Chen, Jing Zong, Zhixuan Huang, Junxin Liu, Qifeng Li

**Affiliations:** College of Precision Instrument and Opto-Electronics Engineering, Tianjin University, Tianjin, China

**Keywords:** laser-induced breakdown spectroscopy, higher density wavelet transform, modified random frog algorithm, infant formula, potassium

## Abstract

Potassium represents one of the most crucial minerals in infant formula that supports healthy growth and development of infants. Here, a novel strategy for the real-time quantification of potassium in infant formula samples is introduced. Using laser-induced breakdown spectroscopy (LIBS) in a data-driven approach, a modified random frog algorithm (MRFA) is adopted in a higher-density discrete wavelet transform (HDWT) domain for the selection of the most important features related to potassium, which is named as DD-LIBS. In DD-LIBS, the HDWT oversamples the LIBS signals in both time and frequency domains by a factor of two, enhancing the spectral expandability in an approximately shift-invariant way. The MRFA is thus capable of isolating the features of potassium with experience accumulated from the collected LIBS data. Such pretreatment combined with a partial least squared (PLS) model can significantly suppress the uncontrolled shift and broadening effects on multivariate calibration, improving the capability of LIBS for accurate quantification of potassium. The present work demonstrates the feasibility of DD-LIBS for the quantification of potassium content of 90 commercial infant formula samples. A satisfactory result illustrates DD-LIBS as a feasible tool for real-time analysis of potassium content with little sample preparation. This strategy may be well extended to other element detection in the presence of uncontrolled interference.

## Introduction

Infant formula, as a breast-milk substitute, plays a significant role since it is the sole source of nutrition for some infants (Deckelbaum et al., 2004; Meucci et al., 2010; Codex, 2015; AOAC International, 2016). The international standard for infant formula set by Codex Alimentarius Commission (CAC) has a strict requirement of the essential composition and nutrition content (Codex, 2015). Meanwhile, all infant formulas marketed must also meet local standards, which are based on the national physique and health level (The Ministry of Health People's Republic of China, 2010b). As an essential cation in intracellular fluid, potassium is one of the most important minerals to support healthy growth and development of infants, because potassium is critically involved with acid-based balance function, osmotic pressure regulation, nerve impulse conduction, muscle construction and Na^+^/K^+^ ATPase (Soetan et al., 2010). An incorrect intake of potassium can also cause diseases (such as hyperkalemia and hypokalemia), which therefore turns the correct control of potassium content of infant formula into a superior importance for both international and local standards (Deckelbaum et al., 2004; Koletzko et al., 2005; The Ministry of Health People's Republic of China, 2010b; Codex, 2015).

To determine the potassium content, the current standard analytical methods are mostly based on atomic absorption spectrophotometry (AAS) (The Ministry of Health People's Republic of China, 2010a), inductively coupled plasma atomic emission spectrometry (ICP-AES) (The Ministry of Health People's Republic of China, 2010a; ISO, 2018a) and inductively coupled plasma mass spectrometry (ICP-MS) (ISO, 2018b), etc. These methods require a laborious and time-consuming sample processing procedure, together with strictly controlled laboratory environment and large sample volume (Panne et al., 2001; Awan et al., 2013; Matsumoto et al., 2016). However, the huge consumption of infant formula at a level of million tons greatly challenges the efficiency of current analytical methods (Tan et al., 2017), and leads to the necessity to develop an efficient and simple method for quantifying the potassium content in infant formula.

Laser-induced breakdown spectroscopy (LIBS), an optical emission spectroscopy technique, presents a potential solution to this challenge (Aragón and Aguilera, 2008). In LIBS, a high-power density laser pulse is focused on a target material in less than a nanosecond, during which a high-temperature plasma is generated by vaporizing a small portion of the target (Zheng et al., 2014). As a result, the radiant characteristics of elements are emitted by the excited atomic, ionic, and molecular fragments produced by the plasma (Harmon et al., 2006; Bousquet et al., 2007). Hence, LIBS offers a strong capability to rapidly detect the element contents in many type of samples (Panne et al., 2001; Bousquet et al., 2007; Hussain and Gondal, 2008; Eseller et al., 2010), with little sample preparation (Hahn and Omenetto, 2010; Hou et al., 2016).

The development of lasers, optics and charge-coupled array detectors has driven a critical revolution in the sensitivity of LIBS, making it a “future superstar” analytical method (Hou et al., 2016). However, the complex process of laser-sample and plasma-particle interactions may distort LIBS peaks (Hahn and Omenetto, 2012). The spectral interference presented in the LIBS signals often leads to an unresolved, broadened and often shifted center of gravity that introduces wavelength shift of spectral peaks (Cremers and Radziemski, 2013), which compromises the LIBS calibration performance. Alternatively, a calibration-free LIBS (CF-LIBS) based on strict theoretical assumptions of laser induced plasma may estimate analyte concentrations correctly. However, CF-LIBS data are severely affected by the self-absorption effect and estimation of plasma temperature (Sun and Yu, 2009), which is challenging for pharmaceutical applications. To improve calibration results, the higher-density discrete wavelet (HDWT) signal processing method with shift-invariant capability becomes a good candidate (Selesnick, 2006). With HDWT, a minor wavelength shift in the raw spectra will not cause a significant variance of the HDWT coefficients at different scales (Qin et al., 2010), which guarantees the reliability of the future calibration models with the HDWT coefficients.

The unique feature of HDWT is that it processes the spectral data in an approximately shift-invariant way, while oversampling the spectral signals in both time and frequency domains by a factor of two, as opposed to the shift-variant downsampling in the conventional discrete wavelet transform (DWT) (Selesnick, 2006). It allows to generate triple wavelet coefficients and thus enables to isolate the localized LIBS spectral features more accurately and robustly (Han et al., 2017). After being processed by HDWT, the LIBS spectral bands of potassium can be well extracted by specific HDWT coefficients, which can be optimized by the feature selection methods (Yun et al., 2013). Since the underlying mechanism of LIBS signals is too complex to be interpreted directly, the observed LIBS data themselves must drive variable selection to optimize multivariate calibration (Parab et al., 2009).

Several feature selection procedures have been developed, including random frog algorithm (RFA) (Li et al., 2012), competitive adaptive reweighted sampling (CARS) (Li et al., 2009), uninformative variable elimination (UVE) and its derivation (Cai et al., 2008; Moros et al., 2008), and randomization tests (Kennedy and Cade, 1996) etc. Among above-mentioned procedures, RFA presents a unique advantage in processing high dimensional spectral data without any prior knowledge that matches the demand of data-driven well. However, the RFA tends to generate a semi-random result that may not correlate accurately with targeted chemicals. In this case, a modified random frog algorithm (MRFA) is adopted by the multiple resampling strategy, in which the RFA has executed hundreds of times to select variables with the highest probability. Therefore, the MRFA is expected to improve the reliability of the LIBS models.

In this work, a data-driven strategy is proposed to isolate the spectral features of potassium with experience accumulated from the observed LIBS data. This strategy aims to estimate the relationship between LIBS spectral datasets and potassium concentrations from the existing input-output data (Gani et al., 2009), which is named as data-driven LIBS (DD-LIBS). In DD-LIBS, the MRFA was adopted in the HDWT domains instead of raw LIBS spectra to avoid spectral interference. A calibration model was then constructed with the selected HDWT coefficients. The DD-LIBS strategy was validated by using 90 commercial infant formula samples.

## Materials and methods

### Sample resource and preparation

Samples of 90 commercially available infant formulas were purchased from the local market, which includes 24 mainstream brands in China. The potassium content was measured by flame atomic absorption spectrometry according to the Chinese national test standard method GB5009.91-2017. To reduce the effects of particle size on LIBS signals, solid infant formula samples were pressed into compact pellets by using a hydraulic press machine under 30 MPa pressure. The measurable characteristics of diameter, thickness, and mass of the pellets were 20 mm, 10 mm, and 4 g, respectively.

### Laser-induced breakdown spectrometry system

In this study, an Ocean Optics LIBS 2500-7 spectrometer system was equipped with CFR Nd. YAG Laser source (LIBS-LAS200MJ, Big Sky Laser Technologies). The laser was operated at a fundamental wavelength of 1,064 nm, and the pulse energy utilized in this experiment was 50 mJ. The pulse duration was 9.5 ns, and the pulse repetition rate was 10 Hz. The LIBS 2500-7 has seven channels to provide a broad spectral wavelength range from 200 to 880 nm, covering the emission spectra of all elements. Each channel is equipped with a 2048-element linear CCD array to present a high optical resolution of 0.1 nm (FWHM). The frame rate was 10 Hz. The integration time was 2.1 ms, and it could be changed in a free-run mode to match sample properties. The trigger delay was from −121 to +135 μs in 500 ns steps. The delay time was set at 0.83 μs, which was determined through optimizing the signal-background ratio (SBR) and characteristic spectral intensity.

### Experimental procedure

For each LIBS analysis, the pellets were put on the sample stage, and 10 different spots of one pellet were evenly selected for LIBS measurement, which reduces the effects of inhomogeneity and surface variations on LIBS signals. Each spot was ablated with 10 laser pulses. As a result, total 100 LIBS spectra were collected and averaged into a single LIBS spectrum, which improves the stability of LIBS experiments.

### Calibration approach

Samples were randomly divided into two sets, i.e., a 65-sample set was used to build a calibration model and a 25-sample set was used to validate the calibration model.

#### Normalization methods

In order to use LIBS in a timely manner, minimal sample pretreatment is preferred. Thus, in LIBS measurement, normalization is performed to compensate for physical variations and sample matrix differences. In this work, five normalization methods, such as average, normalization by norm, spectral area, spectral height, and carbon emission lines (Abdel-Salam et al., 2013; Castro and Pereirafilho, 2016; dos Santos Augusto et al., 2017), were compared.

#### Data analysis through data-driven LIBS

The LIBS spectra are affected by matrix effect and other unknown interference, resulting in broadened and shifted LIBS peaks. DD-LIBS is thus proposed to reduce the effect of peak broadening and shift on multivariate calibration. To correct shifted and expanded spectral peaks, HDWT was applied by implementing the three channel filter banks to conduct an oversampling operation for generating nearly shift-invariant wavelet coefficients.

**Figure 1:**
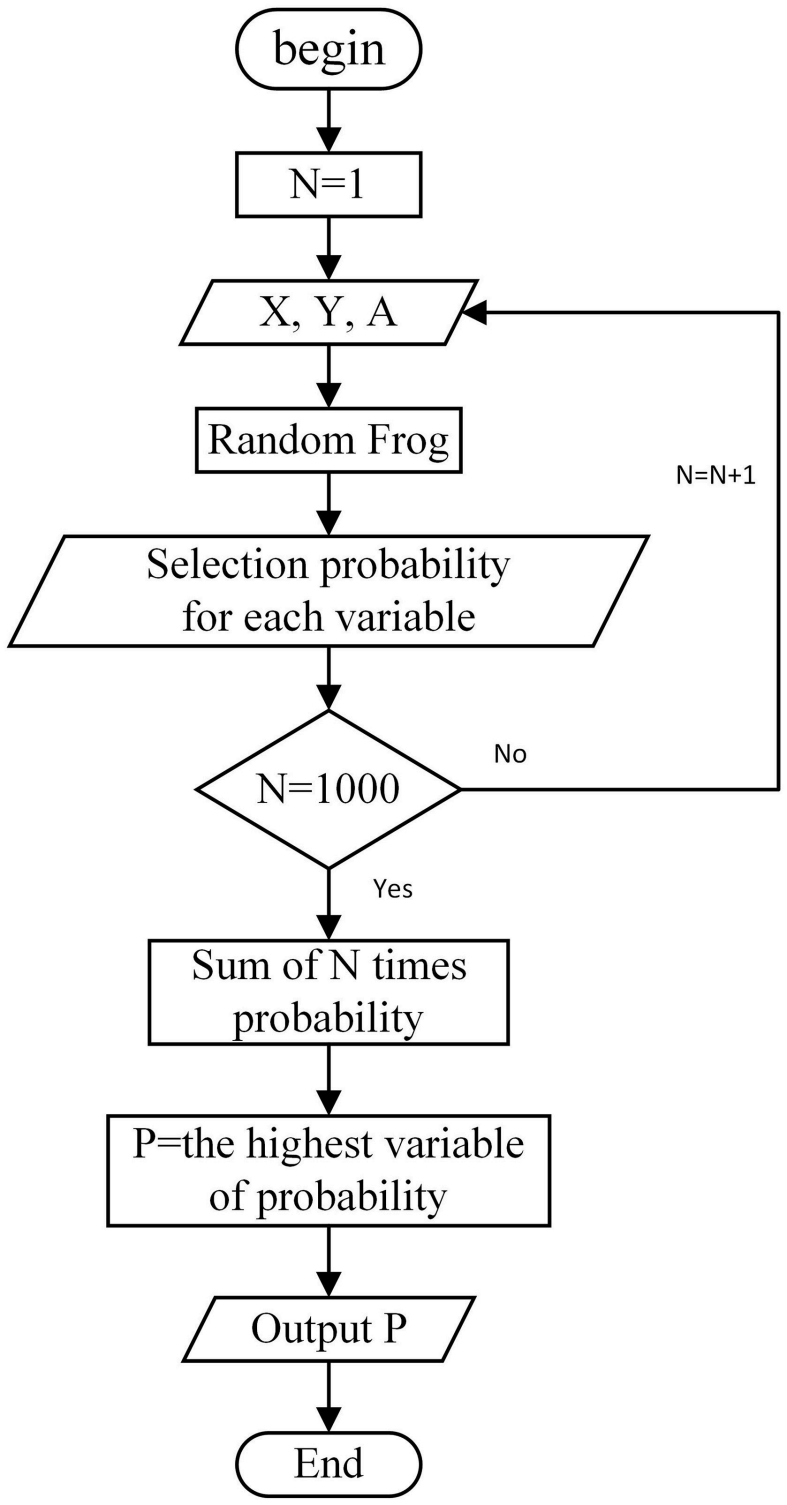
Flowchart of modified random frog algorithm (MRFA), where X is the HDWT coefficients of calibration set, Y is the reference values of calibration samples, A is the number of PLS factors, N is the number of MRFA runs, P is the sum of the selected probability of each variable.

After the HDWT calculation, the raw LIBS spectra were decomposed into localized components labeled by a scale, facilitating the feature selection methods to isolate the spectral bands related to potassium. Then, the MRFA was performed by using the bagging strategy, assigning 70% samples to a training subset and 30% samples to a validation set. The procedure was repeated for 1,000 times to generate 1,000 different selection probabilities of each HDWT coefficient for accumulation. The flowchart of MRFA is shown in Figure 1.

In this work, only the HDWT coefficient with the highest probability was selected for further calibration because it provided valuable robustness against the uncontrolled and unknown spectral interference, and the feature selection result can be easily validated by the reference LIBS spectra of potassium.

As mentioned above, DD-LIBS was established by integrating HDWT, MRFA and PLS together. The HDWT codes were written in Matlab 2013a based on the Selesnick's theory (Selesnick, 2006). The programs of PLS and RFA were available in the libPLS toolbox for Matlab (Li et al., 2014), and the MRFA was modified from RFA in Matlab 2013a.

#### Evaluation parameters

The root mean square error of cross-validation (*RMSECV*) was used to determine the HDWT parameters, and the coefficient of determination (*R*^2^) was used to evaluate the calibration performance of the developed models (Chu, 2011):

(1) $$\begin{array}{r} RMSECV=\sqrt{\frac{\sum_{i=1}^{m}{\left(y_{i,actual}-y_{i,predicted}\right)}^{2}}{m-1}} \end{array}$$

(2) $$\begin{array}{r} R^{2}=1-\frac{\sum_{i=1}^{n}{\left(y_{i,actual}-y_{i,predicted}\right)}^{2}}{\sum_{i=1}^{n}{\left(y_{i,actual}-{\bar{y}}_{i,actual}\right)}^{2}} \end{array}$$

Where *y*_*i, actual*_ is the reference value of the potassium concentration of sample *i, y*_*i, predicted*_ represents the predicted value of sample *i, m* is the number of calibration samples, and ${\overset{-}{y}}_{i,actual}\;$ represents the average reference concentration of all samples. When we obtain a *RMSECV* from the prediction set, we refer it as a *RMSEP*. The evaluation criterion is very simple: the smaller the value of *RMSEP* is, the stronger the prediction capability of the model is.

The limit of detection (LOD) was calculated by using the following equation (ICH Guideline, 2005):

(3) $$\begin{array}{r} \text{LOD}=\frac{3.3\times SD_{blank}}{s} \end{array}$$

Where *SD*_*blank*_ is the standard deviation of the baseline near peaks, and *s* is the slope of the calibration curve.

## Results and discussion

### LIBS spectrum of infant formula

**Figure 2:**
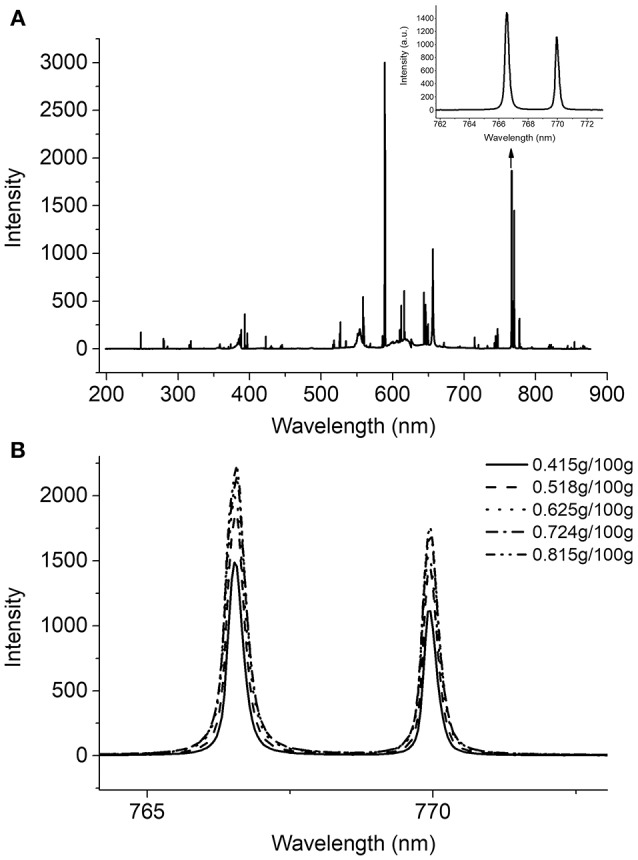
**(A)** A typical LIBS spectrum of infant formula and partially enlarged emission lines of potassium, **(B)** Regional potassium peaks of five samples with different concentrations.

In this work, a typical full spectrum and regional potassium peaks of an infant formula are presented in Figure 2A. The LIBS spectrum of infant formula has sharp characteristic peaks with different intensities, and each peak uniquely corresponds to a specific element. According to the Atomic Spectra Database (ASD) of National Institute of Standards and Technology (NIST), the peaks located at 766.57 and 769.95 nm were selected for quantifying the potassium content in infant formula. As shown in Figure 2B, the spectra of five representative samples with different potassium concentrations were illustrated from 0.415/100 g to 0.815/100 g. It was clear that the intensity of the potassium peaks related to its concentrations accordingly but not linearly, because the potassium peaks were affected by both potassium concentrations and physical parameters (such as laser energy fluctuation and effects related to the sample texture and density). Unfortunately, the contribution of any interference to LIBS was unclear, and DD-LIBS was thus developed to perform the quantitative analysis of potassium by using the existing input-output LIBS data.

### Selection of normalization method

Five normalization methods were compared by calculating the *RMSEP* of each PLS calibration model. The *RMSEPs* of these five normalization methods including average, normalization by norm, spectral area, spectral height, and carbon emission lines, were 0.056, 0.065, 0.076, 0.059, and 0.096, respectively. It is clear that the average normalization strategy was most suitable with the lowest *RSMEP* value and was subsequently applied in this work. After data normalization, the calibration performance of the univariate, PLS and DD-LIBS models was then compared to facilitate the understanding of the LIBS quantification.

### Univariate analysis

**Figure 3:**
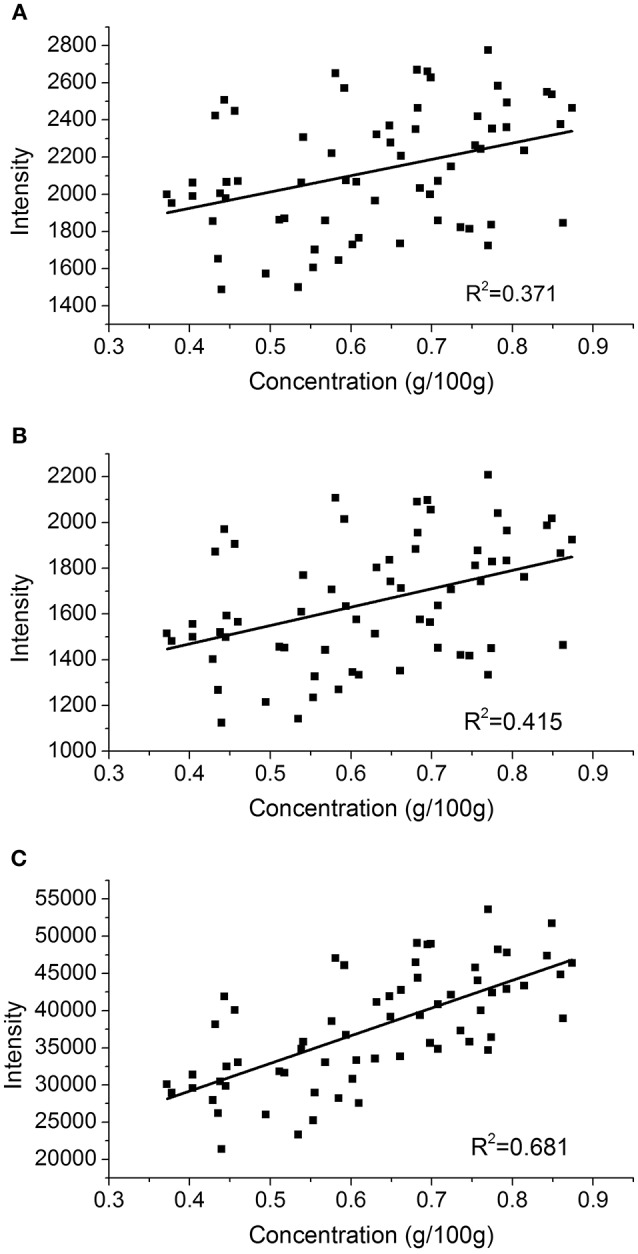
A univariate calibration curve based on **(A)** the intensity of the first peak at 766.57 nm, **(B)** the intensity of the second peak at 769.95 nm and **(C)** the areas of two peaks at 766.57 and 769.95 nm.

The univariate analysis represents the most conventional modeling strategy, in which the analyte's concentration and the peak intensity or the peak area are set as *x* and *y*, respectively (El Haddad et al., 2014). In this work, two calibration curves were made with two potassium peaks as shown in Figures 3A,B. Figure 3C demonstrates another calibration curve using the areas of these two peaks. The LOD obtained from the first peak of potassium was 37 ppm. As shown in Figures 3A,B, the *R*^2^ of both peak height curves are pretty low, which means that the correlation is poor (El Haddad et al., 2014). The *R*^2^ of area (C) is also not satisfactory for quantification even it is slightly higher than the two peaks above-mentioned. The reason is that the univariate analysis is compromised by both matrix effect and sample complexity (Hou et al., 2016; Sanghapi et al., 2016). It is therefore expected that the multivariate analysis could improve the calibration performance through latent projection instead of univariate regression, and PLS was chosen as it is mostly adopted in multivariate calibration.

### PLS calibration

**Figure 4:**
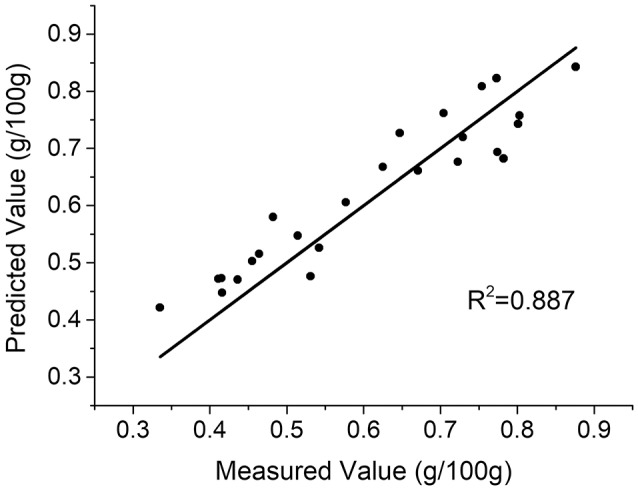
Prediction results of the PLS model with the raw LIBS spectra.

The spectral features of potassium were assigned from 751.90 to 774.86 nm, which contains 512 variables. To evaluate prediction capability of the PLS model, *R*^2^ and *RMSEP* were calculated. Figure 4 demonstrates that the prediction results of the PLS model exceed those of univariate analysis. However, the prediction performance could be further improved through the suppression of the uncontrolled spectra shift and broadening.

### DD-LIBS strategy

In DD-LIBS, the HDWT aims to suppress the effects of peak shift and broadening on multivariate calibration through the oversampling and shift-invariant operation. With the combination of MRFA, DD-LIBS is expected to isolate the spectral features related to the potassium accurately.

#### Determination of HDWT parameters

The performance of HDWT depends on wavelet filters and decomposition scales, which should be optimized before calibration. In HDWT, four wavelet filters with different vanishing moments are available (Selesnick, 2006). Theoretically, the wavelet filter with higher vanishing moment shrinks the peak more efficiently than that with lower vanishing moment (Han et al., 2017). Here, the “bi4” wavelet filter with four vanishing moments was selected, since it possesses the highest vanishing moment in the current HDWT filter bank (Selesnick, 2006). By using the “bi4” filter, the spectral resolution would be expanded by a factor of three, which significantly improved the spectral expandability in an approximately shift-invariant way.

**Figure 5:**
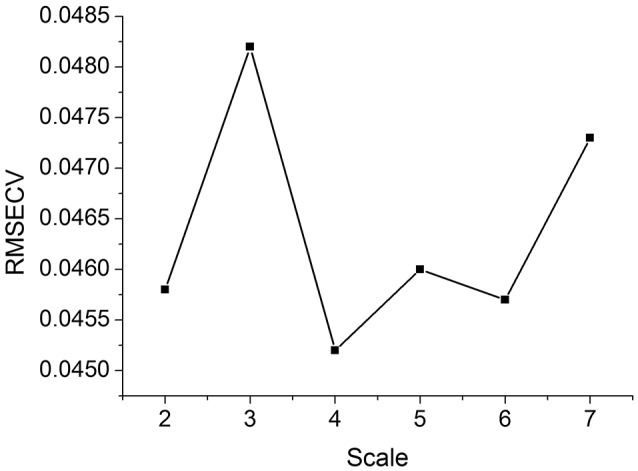
RMSECV of the calibration set with different HDWT decomposition scale parameters.

The decomposition scale is also critical in HDWT, so it was optimized by the minimum *RMSECV* criterion. Figure 5 indicates the relationship between the scale and *RMSECV* using the leave-one-out cross-validation of the calibration set. As a result, the scale four was selected for the HDWT calculation.

#### Feature selection obtained by MRFA

**Figure 6:**
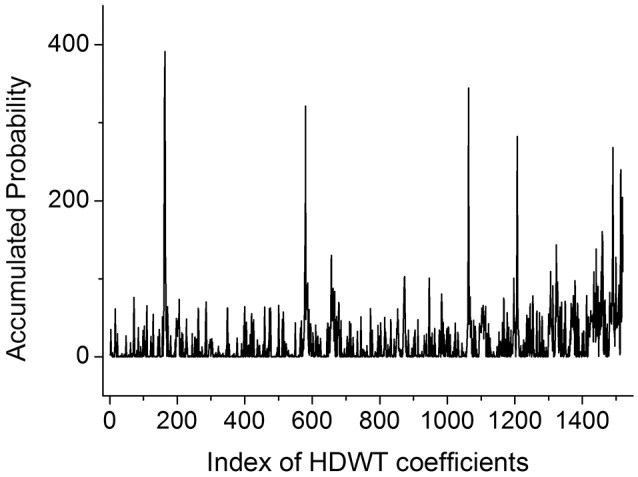
Selection probability of HDWT coefficients obtained from the MRFA.

After the HDWT calculation, the original 512 variables were expanded into 1,520 new variables, providing additional flexibility to isolate the features of potassium in the presence of uncontrolled spectral interference. In the sequence, MRFA was adopted to select the accurate features of potassium. Figure 6 illustrates the accumulated probability of each variable after 1,000 times of MRFA calculation, and the variable with the highest probability was selected for further multivariate calibration.

**Figure 7:**
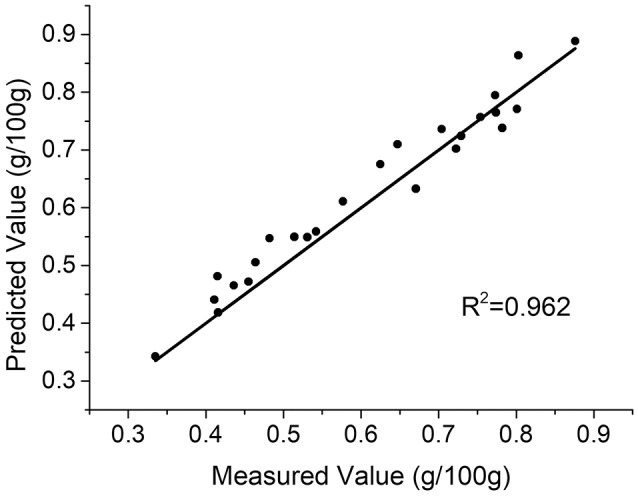
Predicted results of the DD-LIBS model.

With the variables selected by MRFA, a PLS model was built. Only one PLS factor was required for calibration, which reveals that DD-LIBS is capable of isolating the spectral peaks of potassium accurately. As compared to Figure 4, the *R*^2^ of DD-LIBS is improved from 0.887 to 0.962 as shown in Figure 7.

**Figure 8:**
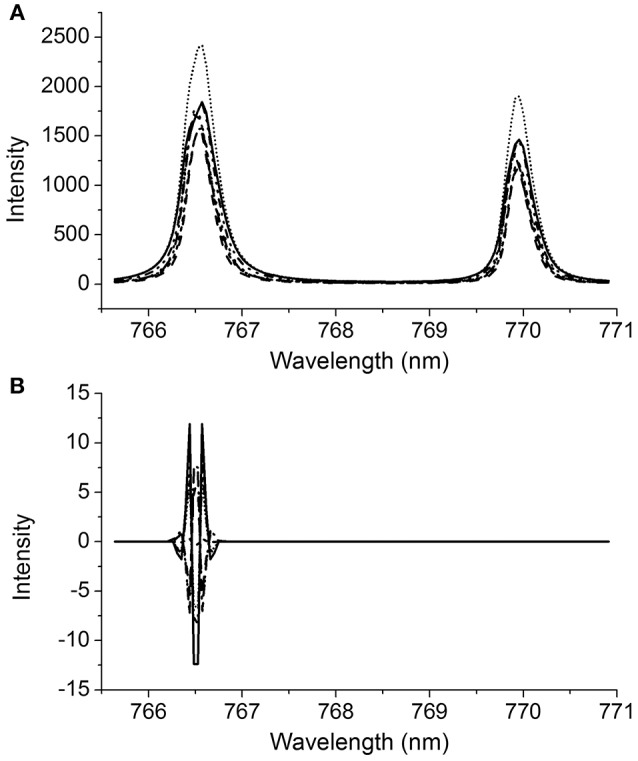
LIBS Spectral information obtained from **(A)** potassium and **(B)** DD-LIBS reconstructed spectra.

It is also of great interest to investigate the reconstructed spectra obtained from the selected variables, which is fundamental to understand how DD-LIBS suppresses the effects of uncontrolled peak shift and broadening on multivariate calibration efficiently. The broadening and shift effect on the LIBS spectral peaks vary from sample to sample as shown in Figure 8A, which may impair the LIBS calibration models. As a comparison, the DD-LIBS filtered data is illustrated in Figure 8B. It is clear that the reconstructed signals of DD-LIBS locate at the same positions as the highest LIBS peak of potassium, and the intensity values at 766.48 and 766.53 nm are the same. It reveals that DD-LIBS cleverly selected the shift-invariant spectral features to overcome the effects of peak shift and peak broadening on multivariate calibration. It is reasonable to expect that DD-LIBS could provide a promising tool to measure potassium content in infant formula accurately, no matter how the uncontrolled interference exists.

### Comparison of different methods

Table 1 shows the prediction results for potassium content in infant formula obtained by different methods. It is obvious that the univariate method presents a poor calibration result, revealing the LIBS spectral analysis should be carefully designed. The PLS model improves the prediction performance of univariate method through multivariate calibration, but the PLS factors are abnormally high. The results illustrate that the additional PLS factors have to be adopted for estimating unknown spectral interference, tending to generate an over-fitting result that relies on the current data set too much. It is unexpected that the combination of RFA and PLS produces a worse result when compared with that of the PLS model. This could be attributed to the effect of spectral interference, e.g., matrix effect, laser energy fluctuation, sample texture and density, and noise, etc. on the feature selection in raw spectra.

**Table 1:** Prediction results for K content in infant formula.

**Methods**	**PLS factor**	**R^2^**	**RMSEP**
Univariate (1st peak)	/	0.099	0.423
Univariate (2nd peak)	/	0.123	0.380
Univariate (area)	/	0.400	0.178
PLS	11	0.887	0.056
RFA-PLS	7	0.882	0.059
HDWT-RFA-PLS	4	0.917	0.050
DD-LIBS	1	0.962	0.036

The HDWT is explored to suppress the spectral interference. The RFA selects the most important HDWT coefficients, resulting in a better prediction precision than that of the RFA-PLS model. As expected, DD-LIBS provides the best prediction results with only one PLS factor, revealing that the LIBS spectral features of potassium are isolated efficiently. As a result, only one PLS factor is required to construct a high-quality calibration model, thus enhancing the reliability and robustness of the LIBS spectral analysis in the presence of uncontrolled interference.

## Conclusion

This study presented a novel strategy, named DD-LIBS, as an approach for real-time quantification of potassium content in commercial infant formula samples. With the combination of HDWT and MRFA, DD-LIBS selected the most important feature related to the potassium accurately, independent of spectral interference. As a result, DD-LIBS generated a high-quality calibration model with only one PLS factor, and the DD-LIBS reconstructed spectra were highly consistent with the original spectral bands of potassium. These satisfactory results suggested a broad expandability of DD-LIBS in the quantification of any targeted element in solid samples in the presence of uncontrolled interference. Once DD-LIBS model has been constructed, it can cleverly predict unknown LIBS spectra as long as these spectra are within a range of relationships learned in the training phase.

## Author contributions

DC planned and supervised the experiments, processed the raw data, revised the manuscript. JZ processed the raw data, wrote the manuscript. JL performed the experiments. ZH advised on data processing and algorithm application. QL revised the manuscript, advised about the principles of LIBS.

### Conflict of interest statement

The authors declare that the research was conducted in the absence of any commercial or financial relationships that could be construed as a potential conflict of interest.

## Abbreviations

LIBS: Laser-induced breakdown spectroscopy
RFA: random frog algorithm
MRFA: modified random frog algorithm
HDWT: higher density wavelet transform
PLS: partial least square.
